# Accidental Ingestion of an Open Safety Pin by a Two-Year-Old Male Child: A Case Report

**DOI:** 10.7759/cureus.82929

**Published:** 2025-04-24

**Authors:** Md Wahiduzzaman Mazumder, Mohammad Rezaul Islam, Fahmida Begum, Nadira Musabbir, Ambia Khatun

**Affiliations:** 1 Pediatric Gastroenterology, Bangabandhu Sheikh Mujib Medical University, Dhaka, BGD

**Keywords:** endoscopic removal, esophagus, foreign body (fb), gastrointestinal (gi), open safety pin

## Abstract

Accidental foreign body (FB) ingestion is common in the pediatric age group between six months and five years. Most ingested FBs pass easily through the esophagus, into the stomach, and are expelled from the body without complications. However, some of these foreign bodies may get stuck in any part of the gastrointestinal (GI) tract, and endoscopic removal may be required to avoid complications. We present a case involving the endoscopic removal of an open safety pin from the duodenum of a two-year-old boy. The safety pin went unnoticed until the mother realized one was missing, leading to an incidental diagnosis.

## Introduction

Ingesting foreign bodies (FB) accidentally is a frequent issue among children [1]. Nearly all FB ingestions occur in children around five years of age or younger due to developmental concerns, behavioral issues, etc. [2, 3]. Approximately 20% of children aged between one and three years have a history of ingesting FBs at some point in their lives [4]. Usually, children explore everything by inserting objects into their mouths, and sometimes they can be swallowed [5, 6]. When FBs get into the gastrointestinal (GI) and respiratory systems, they can cause serious illness and even death, and need to be evaluated immediately. Around 40% of FB ingestions in children happen without being seen, and many of these unnoticed cases don't show symptoms [7]. When children ingest FBs, they may experience a range of symptoms, including vomiting, difficulty breathing (dyspnea), wheezing, restlessness, abdominal distension, and abdominal pain. While FBs can become lodged in any part of the GI tract, they tend to get stuck more frequently in specific areas such as the cricopharyngeal region, the middle third of the esophagus, the lower esophageal sphincter, the pylorus, or the ileocecal valve [8]. Accidental ingestion of sharp and a few non-sharp foreign bodies like safety pins, nails, hairpins, screws, pine needles, fish bones, plastic toys, magnets, coins, button batteries, etc. constitutes an absolute indication of removal due to the high risk of damage to the GI tract and surrounding parenchymatous organs [9]. Major complications following ingestion of sharp FBs have been documented in the literature. The most unusual ones include perforation of the heart by a swallowed safety pin [10], formation of an abscess or fistula [11], and migration of an ingested needle to the pancreas [12]. Endoscopic procedures offer a non-surgical method of removing FBs in pediatric cases. In less than 1% of cases, surgical intervention may be required, whereas about 20% of cases need endoscopic removal [13-15]. To remove FBs during endoscopic procedures, a wide range of endoscopic retrieval devices is available. To manage FB removal effectively, skilled endoscopists must work together and have access to a variety of retrieval devices. We, therefore, present a case of accidental ingestion of an open safety pin found in the third part of the duodenum of a two-year-old male child.

## Case presentation

**Figure 1:**
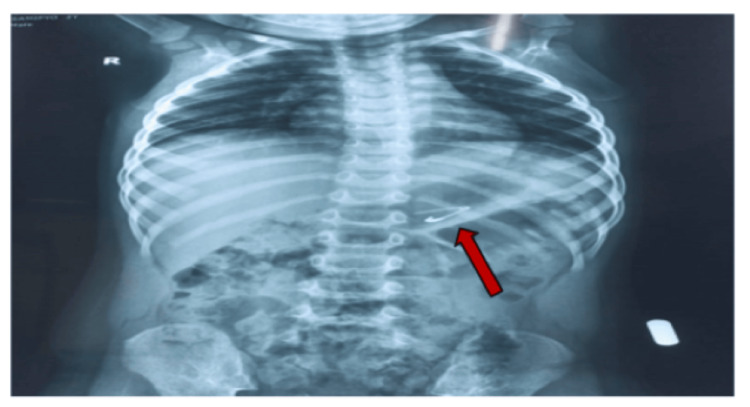
An X-ray of the abdomen showing a foreign body, an open safety pin, in the abdomen.

**Figure 2:**
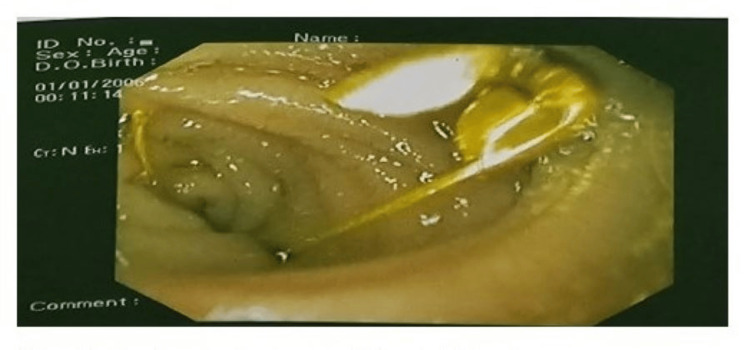
The foreign body is seen stuck in the third part of the duodenum.

**Figure 3:**
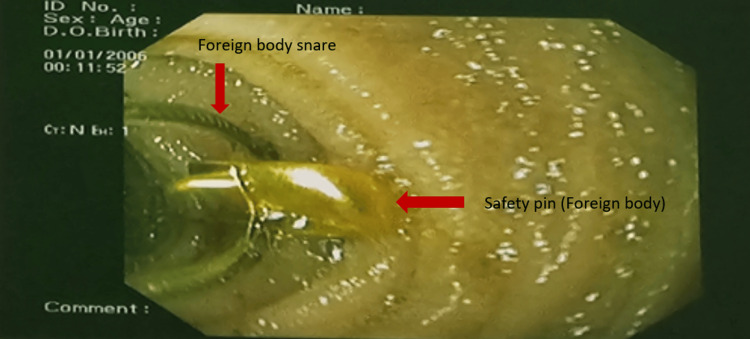
The foreign body being held with a foreign body snare

**Figure 4:**
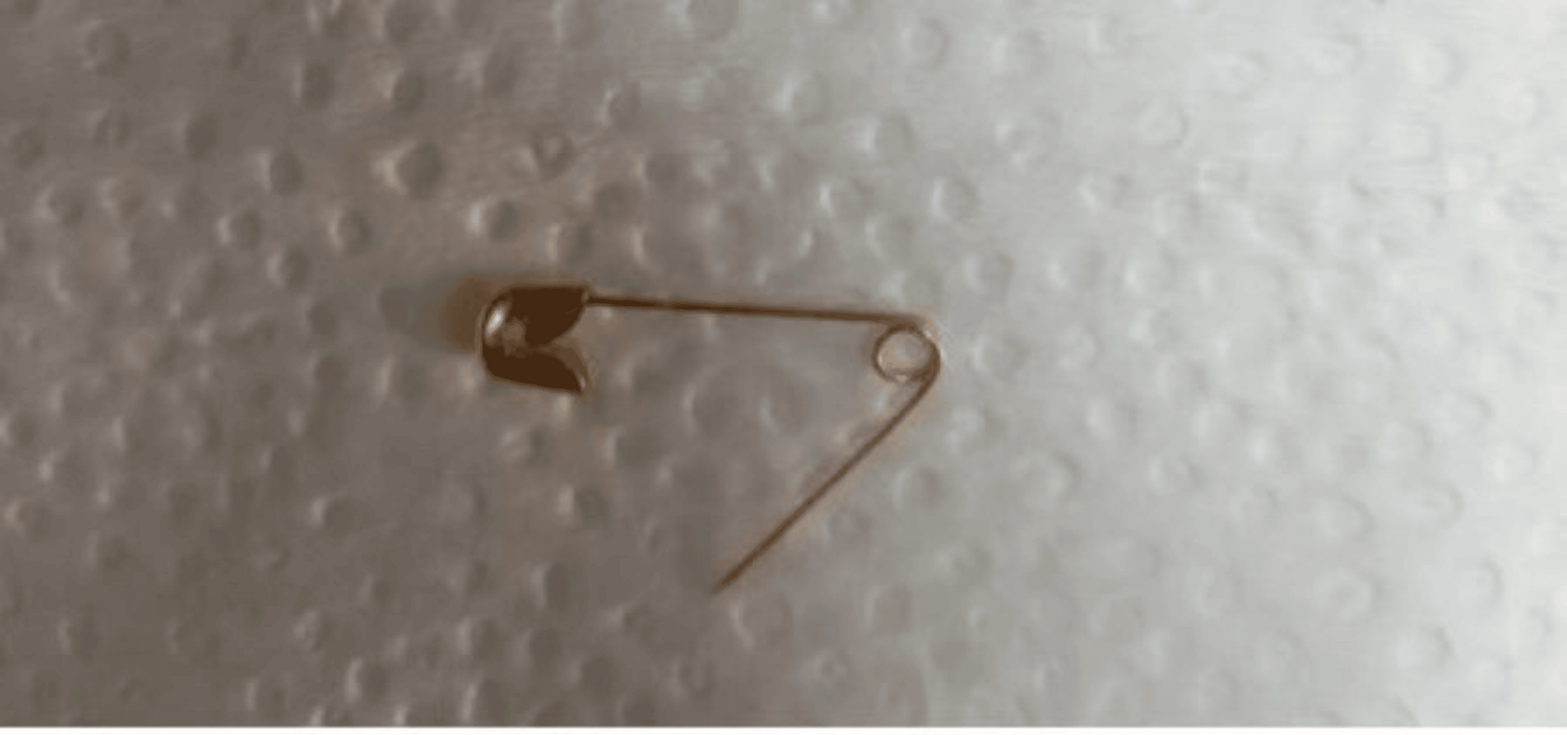
An open safety pin removed

A two-year-old male child presented at the outpatient department of Pediatric Gastroenterology and Nutrition (PGN), Bangabandhu Sheikh Mujib Medical University (BSMMU), Dhaka, Bangladesh, following accidental ingestion of an open safety pin during play. Interestingly, the child remained asymptomatic despite the ingestion. When the mother realized that one safety pin was missing, she suspected that her child might have swallowed it. Following investigations like complete blood count, hepatitis B surface antigen (HBsAg), etc., including an abdominal X-ray, revealed that the safety pin was indeed lodged in the abdomen (Figure 1). A decision was made to perform endoscopic removal of the FB, and the patient was referred to a skilled endoscopist in the Department of PGN, BSMMU. Upon examination, the patient was alert, vitally stable, and had a soft, non-tender abdomen with normal bowel sounds. Other systemic examinations revealed no abnormalities. After obtaining informed written consent, the patient was positioned with a plastic dental bite guard securely in place, and the endoscope was gently inserted into the oropharynx to visualize the esophagus, stomach, and duodenum. The endoscopy revealed that an open safety pin was vertically lodged in the third part of the duodenum (Figure 2). Upon examination, there was no evidence of duodenal ulceration or perforation. Initially, the open safety pin was grasped using an alligator forceps, but unfortunately, it slipped. However, the medical team then successfully secured the safety pin using an FB snare. These remained en bloc, making sure the sharp ends of the free ends dragged back into the stomach with extreme care and precision. The scope, along with the FB snare, was gradually and cautiously pulled to safely retrieve the foreign object (Figure 3). Subsequently, maintaining continuous insufflation, the endoscope, along with the FB snare gripping the open safety pin, was skillfully guided through the gastroesophageal junction, into the esophagus, and then out, ensuring that the sharp ends trailed safely. During the initial attempt, the open safety pin was successfully retrieved (Figure 4). After the successful retrieval of the open safety pin, the endoscope was reintroduced to assess any potential damage to the esophageal, stomach, and duodenal mucosa resulting from the extraction. Fortunately, no new injuries were observed. The patient was subsequently discharged and followed up for a few hours, during which there were no reported complications.

## Discussion

It’s quite common for children to accidentally ingest foreign objects. Among the most frequently ingested items are coins, followed by toys, toy parts, magnets, batteries, safety pins, screws, marbles, bones, and even food boluses [6, 16]. Soft-ended FBs do not cause any issues. However, those with sharp ends cause major issues. An open safety pin with one sharp, pointy end was consumed by the patient in this instance (Figure 2). The patient may be asymptomatic, like in this case, or may present with symptoms like drooling, hyperventilation, dysphagia, odynophagia, and refusal of feeds. When children remain asymptomatic at first, the common areas of possible FB impaction are the pylorus, duodenal curve, and ileocecal valve. Complications such as intestinal perforation or fistula formation may later present in <1% of cases [1].

The most common location of FB impaction is the esophagus, which is also the narrowest section of the GI tract. The most frequent location of FB impaction in the esophagus is the thoracic inlet, which is followed by the gastroesophageal junction and the aortic arch [16]. Foreign bodies larger than 2 cm in diameter have a lower chance of passing through the pylorus, and bodies longer than 6 cm run the risk of getting stuck at the duodenal curve or the pylorus [16]. The risk of impaction is very low if the foreign body goes through the stomach. Extremely unlikely, a large or sharp foreign body lodged in the rectum, appendix, duodenum, pylorus, or a site of acquired or congenital GI tract narrowing [17]. In this instance, the FB was lodged vertically in the third portion of the duodenum (Figure 2). A proficient endoscopist can remove an ingested FB endoscopically with a high success rate, minimal morbidity, and minimal mortality [18]. Eighty-five adults and 2,000 children were found to have died in a study that included two large series reports [19]. For optimal FB removal, specialized forceps such as alligator or rat tooth forceps are recommended. These tools are equipped with an overtube or a latex rubber hood to safeguard the pharyngeal and esophageal mucosa from damage [8]. We employed an FB removal snare in this instance. During follow-up visits, no complications were noted; these findings were consistent with a retrospective study conducted by Kramer et al. [20]. In 96.9% of cases, endoscopic FB removal was successful, and only 3.1% of cases required surgical intervention, according to the study's authors. Mucosal laceration and suspected perforation were the complications noted in 6.9% of cases; all of these cases were treated conservatively [20].

## Conclusions

Using pediatric and ancillary endoscopic equipment, a skilled endoscopist can effectively manage FB ingestion in the upper gastrointestinal tract in children. The type of ingested FB, the children's age, anticipated challenges, and emergencies must all be taken into account prior to an endoscopic procedure.
